# Hidden diversity and phylogeographic history provide conservation insights for the edible seaweed *Sargassum fusiforme* in the Northwest Pacific

**DOI:** 10.1111/eva.12455

**Published:** 2017-02-19

**Authors:** Zi‐Min Hu, Jing‐Jing Li, Zhong‐Min Sun, Xu Gao, Jian‐Ting Yao, Han‐Gil Choi, Hikaru Endo, De‐Lin Duan

**Affiliations:** ^1^ Key Laboratory of Experimental Marine BiologyInstitute of OceanologyChinese Academy of SciencesQingdaoChina; ^2^ Laboratory for Marine Biology and BiotechnologyQingdao National Laboratory for Marine Science and TechnologyQingdaoChina; ^3^ College of Earth ScienceUniversity of Chinese Academy of SciencesBeijingChina; ^4^ Laboratory of Marine Organism Taxonomy & PhylogenyInstitute of OceanologyChinese Academy of SciencesQingdaoChina; ^5^ Research Centre for Inland SeasKobe UniversityRokkodaiKobeJapan; ^6^ Faculty of Biological Science and Research Institute for Basic ScienceWonkwang UniversityIksanKorea; ^7^ Faculty of FisheriesKagoshima UniversityKagoshimaJapan; ^8^ Present address: Institute of Marine BiologyCollege of OceanographyHohai UniversityNanjing210098China

**Keywords:** Asia–Northwest Pacific, biodiversity conservation, climate change, cryptic lineage, phylogeographic process, *Sargassum fusiforme*

## Abstract

Understanding the evolutionary processes that have created diversity and the genetic potential of species to adapt to environmental change is an important premise for biodiversity conservation. Herein, we used mitochondrial *trn*W‐L and *cox*3 and plastid *rbc*L‐S data sets to analyze population genetic variation and phylogeographic history of the brown alga *Sargassum fusiforme*, whose natural resource has been largely exterminated in the Asia–Northwest Pacific in the past decades. Phylogenetic trees and network analysis consistently revealed three major haplotype groups (A, B, and C) in *S. fusiforme*, with A and B distributed in the Japan‐Pacific coast. Group C consisted of three subgroups (C1, C2, and C3) which were distributed in the Sea of Japan, the Yellow–Bohai Sea, and East China Sea, respectively. Isolation‐with‐migration (IM a) analysis revealed that the three groups diverged approximately during the mid‐Pleistocene (*c*. 756–1,224 ka). Extended Bayesian skyline plots (EBSP) showed that groups A and B underwent relatively long‐term stable population size despite a subsequent rapid demographic expansion, while subgroups C2 and C3 underwent a sudden expansion at *c*. 260 ka. *F*_ST_ and AMOVA detected low population‐level genetic variation and high degrees of divergence between groups. The cryptic diversity and phylogeographic patterns found in *S. fusiforme* not only are essential to understand how environmental shifts and evolutionary processes shaped diversity and distribution of coastal seaweeds but also provide additional insights for conserving and managing seaweed resources and facilitate predictions of their responses to future climate change and habitat loss.

## 1 Introduction

Population genetic differentiation and biogeographic regionalization, as the central issues in conservation biology, have been well illustrated to directly relate to evolutionary forces such as isolation, mutation, and historical demography (Dufresnes et al., 2013; Millar & Byrne, 2013). Thus, understanding phylogeographic processes that determine genetic diversity and distribution patterns of lineages over time and space has become a fundamental goal of conservation biogeography (Richardson & Whittaker, 2010). On the one hand, phylogeographic lineages within species constitute ecologically and evolutionarily significant units (ESUs) for conservation and management (D'Amen, Zimmermann, & Pearman, 2013; Dufresnes et al., 2013). On the other hand, evolutionary processes at either macro‐ or microgeographic scale can recreate, maintain, and restore specific phenotypic or genotypic variants—the priority goals of conservation planning (Crandall, Bininda‐Emonds, Mace, & Wayne, 2000; Moritz, 1999). Changing climate may impact species ranges, biodiversity, and evolutionary legacy (Lavergne, Mouquet, Thuiller, & Ronce, 2010). Therefore, it is crucial to characterize the genetic lineages that may reflect the adaptive potential of species and may be relevant to future response to environmental change.

The Asia–Northwest Pacific (ANP) is an important marine center harboring rich seaweed diversity and endemism in the world's oceans (Keith, Kerswell, & Connolly, 2014; Kerswell, 2006). On the coasts of mainland East Asia, there are several well‐collected regions (e.g., China and Korea) where around 500 species of seaweed have been recorded (Norton, Melkonian, & Andersen, 1996; Tseng, 1983). In the Japanese Archipelago, similar collecting efforts have documented 1,510 seaweed species which are two to three times more than those in regions with similar latitude ranges (e.g., S. Alaska–Oregon and N. British Isles–Morocco) (Norton et al., 1996). However, the distribution pattern of seaweed richness in the ANP, particularly around Japan and China, has been largely destroyed by environmental shifts in the past few decades (Sun, Ning, Le, Chen, & Zhuang, 2010; Tanaka, Taino, Haraguchi, Prendergast, & Kiraoka, 2012). For instance, the Nanji Island, China, underwent a rise in annual sea surface temperature (SST) by 0.5°C during 1960–2006, with the species number of cold‐temperate seaweeds reduced from 7 in 1959 to 2 in 2006 (Sun et al., 2010). The rapidly decreased seaweed diversity imposes an urgent need to manage and conserve seaweed resources and biodiversity under accelerating environmental changes.

**Figure 1:**
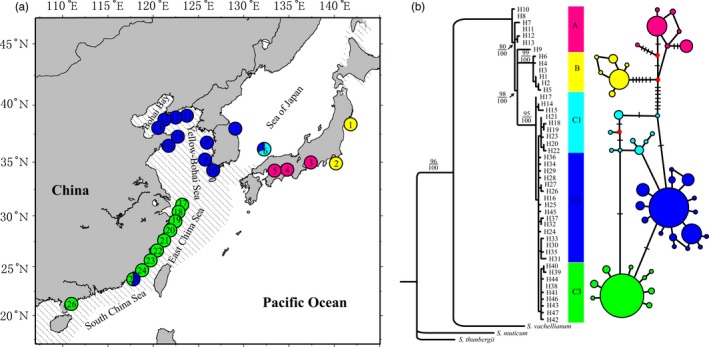
Haplotype distribution pattern (a) and maximum‐likelihood (ML, upper)/Bayesian inference (BI, lower) (b) inferred from mtDNA *trn*W‐L+*cox*3 data. Shaded sea areas are continental shelves that would have been exposed to the air during periods of low sea level. Numbers at tree nodes are percentages of bootstrap support. Each line between main haplotypes represents one mutation step. Detailed locality information is shown in Table 1

*Sargassum fusiforme* (Harvey) Setchell is a large brown alga found in the lower intertidal zone throughout southern Japan, the rim of the Yellow–Bohai Sea, and East China Sea (Figure 1). Ecologically, this species can form dense beds and make substantial contribution to coastal marine ecosystems by acting as primary producer, providing spawning, nursery, and feeding grounds for fish and invertebrates, and improving environmental conditions (e.g., water motion and temperature) (Hwang, Yoo, Baek, & Park, 2015). Socioeconomically, *S. fusiforme* is widely utilized as food, polysaccharide resource, and medical agent (Mao, Li, Gu, Fang, & Xing, 2004; Zhu, Heo, & Row, 2010). Its market value in Japan was estimated to be 130 billion JPY during the late 1990s (Murata & Nakazoe, 2001). The large market demand caused extensive harvest of *S. fusiforme* in nature, with an annual production of 8,000–10,000 tons in Japan (Fresh weight) (Ito, 2012) and 32,000 tons in China in 2007 (Pang, Shan, Zhang, & Sun, 2008). Profit‐induced catastrophic harvest, together with habitat degradation, resulted in significant reduction in natural *S. fusiforme* resource in the ANP. As expected, *S. fusiforme* in Rongcheng, China, experienced large‐scale contraction during 1982–2006, with the distribution range declined from 89 to 33 ha, average biomass declined from 886.84 to 210 g/m^2^, and annual production declined from 559 to 7.74 tons (Zhang & Liu, 2009).

Apart from commercial harvest and habitat loss, long‐term changes in regional SST also have generated serious impacts on *S. fusiforme* on the coast of southern Japan and China. It is estimated that the SST rose by 1–2°C along Kagoshima, Japan, in the past four decades (Tsuchiya, Sakaguchi, & Terada, 2011) and the average SST of Kyushu Island increased by 1.2°C during 1900–2010 (Japan Meteorological Agency 2011), leading to a massive reduction in the distribution range and biomass of *S. fusiforme* at marginal areas (Kokubu et al., 2015). In Nanji Island, the rising SST caused *S. fusiforme*–*S. thunbergii* (Mertens ex Roth) Kuntze codominated beds in zonal community in 1959 to become dominated solely by *S. thunbergii* in 2006 (Sun et al., 2010). The contraction of the distribution range and loss of production of *S. fusiforme* in the ANP thus raise an essential question of how to practice efficient measures to conserve this commercially important seaweed species.

The ecological and commercial importance of *S. fusiforme* has stimulated many studies focused on ecophysiological responses to abiotic factors, reproduction modes, and marine cultivation (Ji & Tanaka, 2002; Kokubu et al., 2015; Pang et al., 2008; Zou, Gao, & Ruan, 2006). Although the characterization of intraspecific diversity and phylogeographic structure is fundamental to the conservation and management of species (Newton, Allnutt, Gillies, Lowe, & Ennos, 1999), a comprehensive attempt has yet to be carried out across the range of *S. fusiforme*. A recent molecular survey using mitochondrial *cox*1 revealed that oceanic currents drove asymmetric genetic exchange between *S. fusiforme* populations in the ANP (Hu, Zhang, Lopez‐Bautista, & Duan, 2013), yet the cryptic lineage diversity and evolutionary patterns remain largely unresolved. From a conservation genetic perspective, the failure to survey population genetic structure of *S. fusiforme* may result in overexploitation or localized extirpation of uncharacterized biodiversity (Hueter, Heupel, Heist, & Keeney, 2005). Deciphering the pattern and degree of population subdivision and structured lineage diversity becomes a prerequisite for conserving and managing the *S. fusiforme* resource.

In this study, our main goals were as follows: (i) to quantify the phylogeographic structure and large‐scale assessment of genetic variation within and between populations by integrating mitochondrial and plastid loci, (ii) to detect the historical demography and geographic distribution of lineage/group diversity in the natural range, and (iii) to place current patterns of genetic diversity and phylogeographic structure into both historical and conservation context with the aim of sustaining natural seaweed resources in the ANP.

## 2 Materials and Methods

### 2.1 Sample collection, DNA extraction, and amplification

A total of 586 *Sargassum fusiforme* individuals were collected from 26 sites in the ANP ranging from Ishinomaki, Miyagi, Japan (38.35°N), to Naozhou, Guangdong, China (20.85°N) (Figure 1, Table 1). At each location, 8–34 individuals were randomly sampled with an interval transect >10 meters. Leaf tips of 3–5 cm were dried and stored in silica gel for molecular analysis. Total genomic DNA was extracted using Plant Genomic DNA Extraction Kit (Tiangen Biotech. Co. Ltd., Beijing) or the method developed previously by Hu, Zeng, Wang, Shi, and Duan (2004). The mitochondrial tRNA W‐L spacer (*trn*W‐L) and cytochrome oxidase III subunit (*cox*3), and the plastid spacer intervening the large and the small subunit of ribulose bisphosphate carboxylase oxygenase (RuBisCo *rbc*L‐S), were targeted due to their effectiveness in revealing phylogeographic structure of the congeneric *Sargassum* species (Cheang, Chu, & Ang, 2010; Hu et al., 2011; Li et al., 2016). To improve PCR amplification and sequencing efficiency, we developed new primer pairs for *trn*W‐L and *cox*3 based on the newly available mitochondrial genome sequence of *S. fusiforme* (GenBank accession no. KJ946428): YC3F (5’‐GAAGGGGTGACTGAGGGGTTG‐3’) and YC3R (5’‐AAACTTTATACTTTATTTAGGGGTC‐3’) for *cox*3 with a product length of 724 bp; YTRNF (5’‐ACCCCTGTAGTTATGAAATGAGAAAGTC‐3’) and YTRNR (5’‐ACCCCTACCTCATAGAAGACTGGAA‐3’) for *trn*W‐L with a product length of 649 bp. Plastid *rbc*L‐S was amplified using 3F (5’‐CATCGTGCTGGTAACTCTAC‐3’; Phillips, Smith, & Morden, 2005) and S97R (5’‐CATCTGTCCATTCWACACTAAC‐3’; Peters & Ramírez, 2001) with a length of 795 bp. PCR profile included denaturation at 95°C for 5 min; denaturation at 94°C for 45 s, annealing at 50°C for 1 min, and extension at 72°C for 1 min, 35 cycles; extension at 72°C for 5 min. Electrophoresis, purification, and sequencing were conducted following our previous protocols (Hu et al., 2007, 2011).

**Table 1:** Diversity indices of *Sargassum fusiforme* populations inferred from mitochondrial *Trn*W‐M+*cox*3 and plastid *rbc*L‐S

Sampling localities (Abbreviation)	Coordinates	*Trn*W‐M+*cox*3		*rbc*L‐S			
		*n*/*N*_h_	*h*	π (×10^−2^)	*n*/*N*_h_	*h*	π (×10^−2^)
1. Ishinomaki, Miyagi, Japan (JIS)	38.35°N, 41.43°E	16/5	0.700 ± 0.080	0.065 ± 0.054	16/1	0.000 ± 0.000	0.000 ± 0.000
2. Tateyama Bay, Chiba, Japan (JTA)	35.00°N, 39.83°E	30/2	0.186 ± 0.088	0.014 ± 0.020	30/3	0.130 ± 0.0821	0.025 ± 0.035
3. Chita, Aichi, Japan (JCH)	34.71°N, 36.92°E	19/1	0.000 ± 0.000	0.00 ± 0.000	20/2	0.100 ± 0.088	0.012 ± 0.025
4. Awaji Island, Hyogo, Japan (JAW)	34.28°N, 34.80°E	24/3	0.163 ± 0.099	0.036 ± 0.036	30/4	0.395 ± 0.100	0.058 ± 0.059
5. Naruto, Tokushima, Japan (JNA)	34.24°N, 34.59°E	16/4	0.592 ± 0.122	0.053 ± 0.047	20/2	0.337 ± 0.110	0.042 ± 0.049
6. Ama, Shimane, Japan (JAM)	36.01°N, 32.58°E	26//11	0.871 ± 0.040	0.140 ± 0.092	29/2	0.192 ± 0.090	0.024 ± 0.035
7. Sokcho, Gangwon‐do, Korea (KSO)	38.20°N, 28.59°E	15/2	0.133 ± 0.112	0.010 ± 0.017	14/1	0.000 ± 0.000	0.000 ± 0.000
8. Guido, Chungcheongnam‐do, Korea (KGA)	36.67°N, 26.07°E	8/2	0.250 ± 0.180	0.018 ± 0.026	8/1	0.000 ± 0.000	0.000 ± 0.000
9. Yeongsando, Jeollanam‐do, Korea (KYE)	34.65°N, 25.47°E	19/2	0.281 ± 0.116	0.020 ± 0.026	19/1	0.000 ± 0.000	0.000 ± 0.000
10. Gwanmaedo, Jeollanam‐do, Korea (KGW)	34.23°N, 26.06°E	10/2	0.200 ± 0.154	0.015 ± 0.022	10/1	0.000 ± 0.000	0.000 ± 0.000
11. Yingzuishi, Liaoning, China (CYZ)	39.01°N, 22.73°E	26/6	0.739 ± 0.055	0.079 ± 0.060	24/3	0.236 ± 0.109	0.039 ± 0.047
12. Daquan, Liaoning, China (CDA)	39.04°N, 22.72°E	23/3	0.379 ± 0.117	0.029 ± 0.031	26/4	0.455 ± 0.110	0.063 ± 0.062
13. Beihuangcheng, Yantai, China (CBH)	38.38°N, 20.90°E	29/3	0.305 ± 0.101	0.023 ± 0.027	29/3	0.197 ± 0.095	0.042 ± 0.048
14. Daqin, Yantai, China (CDQ)	38.27°N, 20.85°E	20/2	0.100 ± 0.088	0.007 ± 0.014	24/2	0.083 ± 0.075	0.011 ± 0.023
15. Chengshantou, Weihai, China (CCS)	37.39°N, 22.71°E	28/2	0.071 ± 0.065	0.005 ± 0.012	30/2	0.067 ± 0.061	0.008 ± 0.020
16. Huidao, Weihai, China (CHD)	36.73°N, 21.60°E	26/4	0.222 ± 0.106	0.022 ± 0.027	27/2	0.205 ± 0.095	0.026 ± 0.036
17. Gouqi Island, Zhejiang, China (CGQ)	30.63°N, 22.47°E	12/1	0.000 ± 0.000	0.000 ± 0.000	12/1	0.000 ± 0.000	0.000 ± 0.000
18. Shengsi, Zhejiang, China (CSS)	30.42°N, 22.46°E	24/1	0.000 ± 0.000	0.000 ± 0.000	24/2	0.083 ± 0.075	0.011 ± 0.023
19. Zhujiajian, Zhejiang, China (CZJ)	29.90°N, 22.43°E	13/1	0.000 ± 0.000	0.000 ± 0.000	13/1	0.000 ± 0.000	0.000 ± 0.000
20. Zhumen, Zhejiang, China (CZM)	28.85°N, 21.66°E	14/1	0.000 ± 0.000	0.000 ± 0.000	15/1	0.000 ± 0.000	0.000 ± 0.000
21. Luxi Island, Zhejiang, China (CLX)	27.98°N, 21.16°E	21/4	0.271 ± 0.124	0.027 ± 0.030	22/2	0.173 ± 0.101	0.022 ± 0.033
22. Nanji Island, Zhejiang, China (CNJ)	27.50°N, 21.08°E	32/2	0.272 ± 0.089	0.020 ± 0.025	34/4	0.401 ± 0.096	0.054 ± 0.056
23. Lianjiang, Fujian, China (CLJ)	26.42°N, 19.92°E	31/2	0.280 ± 0.090	0.020 ± 0.025	31/2	0.361 ± 0.084	0.045 ± 0.050
24. Putian, Fujian, China (CPT)	25.25°N, 19.67°E	28/3	0.204 ± 0.098	0.015 ± 0.021	30/3	0.301 ± 0.102	0.040 ± 0.046
25. Dongshan Bay, Fujian, China (CDS)	23.68°N, 17.48°E	24/3	0.562 ± 0.047	0.044 ± 0.040	27/1	0.000 ± 0.000	0.000 ± 0.000
26. Naozhou, Guangdong, China (CNZ)	20.85°N, 10.56°E	26/3	0.219 ± 0.103	0.016 ± 0.022	28/2	0.147 ± 0.089	0.020 ± 0.033

*n*, number of sequences; *N*_h_, number of haplotypes; *h*, haplotype diversity; π, nucleotide diversity.

### 2.2 Molecular diversity, phylogeny, and principal component analysis (PCoA)

Sequences were aligned with MEGA 5.0 (Tamura et al., 2011). Genetic diversity was estimated by the number of polymorphic sites (S), haplotype distribution, number of haplotypes (*N*h), haplotype diversity (*h*), and nucleotide diversity (π), which were all calculated in arlequin 3.5 (Excoffier & Lischer, 2010). To evaluate the relationships among haplotypes, a parsimony median‐joining network was generated with the program network 4.51 (Bandelt, Forster, & Röhl, 1999). JMODELTEST 0.1.1 (Posada, 2008) was used to identify the best substitution model for each locus under the Bayesian information criterion (BIC) (*trn*W‐L+*cox*3: HKY+G, G = 0.1901; *rbc*L‐S: HKY+I, I = 0.7298). The *trn*W‐L+*cox*3 and *rbc*L‐S sequences were applied for phylogenetic analysis, performed using the neighbor‐joining (NJ), maximum‐likelihood (ML), and Bayesian inference (BI) in MEGA 5.0, phyML 3.0 (Guindon et al., 2010), and mrbayes 3.2 (Ronquist et al., 2012), respectively. In the NJ and ML analyses, trees were tested by bootstrapping method with 1,000 replications. For BI analysis, Bayesian searches included four chains. Each chain was run for two million generations with a tree sampling frequency of every 200 generations, with the first 10% of the resulting trees discarded as burn‐in. The congeneric species *S. vachellianum* Greville (GenBank accession no. KR132242), *S. thunbergii* (no. KP280065), and *S. muticum* (Yendo) Fensholt (no. KJ938301) were chosen as out‐groups.

Plastid *rbc*L‐S and concatenated mitochondrial *trn*W‐L+*cox*3 were used, respectively, to test genetic distinctiveness of populations using principal component analysis (PCoA) conducted in GENALEX (Peakall & Smouse, 2012). The conventional population *F*_ST_ comparisons were measured for both mitochondrial and plastid loci to evaluate possible levels of genetic differentiation. All results for significance of covariance components were tested using 10^5^ permutations. Analysis of molecular variance (AMOVA) was also conducted in arlequin to assess the spatial partitioning of genetic variance among the groups defined above.

### 2.3 Dating divergence time

The isolation‐with‐migration model implemented in Ima2 (Hay & Nielsen, 2007; Nielsen & Wakeley, 2001) was used to estimate migration rates (*m*), population sizes (Θ), and divergence time (*t*) based on the concatenated *trn*W‐L and *cox*3 data sets (running as independent loci because they exhibited substantially different mutation rates [Chan et al., 2013]). Ima can incorporate multiple genetic loci independently with locus‐specific mutation scalars. One way to implement such scalars is to pick one locus as a standard with a mutation rate scalar of one and to have the scalars for other loci vary as parameters to be estimated. Specifically, Ima jointly estimates these genetic parameters in a Bayesian sampling framework by calculating posterior probabilities for parameters across a set of likely gene trees (Nielsen & Wakeley, 2001). Several runs were conducted until stationary results were achieved. The final runs included 100 coupled Markov chains, a burn‐in period of 300,000 steps, and a geometric heating model, with the first and second heating parameters of 0.99 and 0.90 for individual chains, respectively. A total of 90,000 genealogies were sampled to estimate the joint posterior probability distributions of the migration parameters. It should be noted that here, we just tried to estimate the relative divergence times between genetic groups defined by phylogenetic analyses because mutation rates are time dependent over evolutionary timescales (Ho et al., 2011). In the Ima analysis, we set both the mutation scalars and mutation rate (the average value of mutation range of mtDNA Cox3, 0.0010–0.0016 site^−1^ Myr^−1^ [Chan et al., 2013]) in the input files. The generation time, *g* (1 year), was applied to convert the output into units of years.

The relative divergence time between groups was also calculated using the equation *d*A = 2μ*T*, where μ is the average substitution rate per site of gene and *d*A is the net average genetic distance between groups (Nei & Li, 1979). The net genetic distance between groups was measured for multiple hits using the equation *d*A = *d*XY—(*d*X + *d*Y)/2 with 1000 replicates in MEGA 5.1 (Tamura et al., 2011), where *d*XY is the mean distance between groups X and Y, and *d*X and *d*Y are mean genetic distance within each group.

### 2.4 Historical demography

Three neutrality tests, the Tajima's *D* (Tajima, 1989), Fu's *F*s (Fu, 1997), and a mismatch distribution approach, implemented in ARLEQUIN 3.5, were used to detect departures from mutation–drift equilibrium that would be indicative of changes in historical demography and natural selection. Current (θ_π_) and historical (θ_W_) genetic diversities were assessed using DNASP 5.0 (Librado & Rozas, 2009). Comparing these two estimates can provide insight into population dynamics, including recent bottlenecks (if θ_π_ < θ_W_) or recent population growth (if θ_π_ > θ_W_) (Pearse & Crandall, 2004; Templeton, 1993).

Extended Bayesian skyline plots (EBSPs) were produced in BEAST 1.7.4 (Drummond & Rambaut, 2007; Heled & Drummond, 2008) to estimate the pattern of population growth with combined mitochondrial and plastid loci. Mitochondrial *trn*W‐L, *cox*3, and plastid *rbc*L‐S groups were analyzed using the HKY+G, HKY+G, and HKY+I substitution model separately, with empirical base frequencies. We chose to use a strict molecular clock with a substitution rate of 0.0010–0.0016 M year^−1^ for *cox*3 and the default estimated rate of 1.0 for *trn*W‐L and *rbc*L‐S, and a stepwise skyline model initiated with the UPGMA tree. The MCMC parameters were set as follows: 9 × 10^8^ iterations, sampling every 9000 iterations, and the first 9 × 10^7^ iterations discarded as burn‐in.

## 3 Results

### 3.1 Genetic diversity and haplotype distribution

Concatenated mitochondrial data sets were obtained from 560 individuals with an aligned length of 1373 bp, including 649 bp of *trn*W‐L and 724 bp of *cox*3. The *trn*W‐L yielded 22 haplotypes (GenBank accession numbers: KX085175‐KX085196), and *cox*3 yielded 26 haplotypes (GenBank accession numbers: KX085149‐KX085174). The concatenated *trn*W‐L+*cox*3 alignment consisted of 50 polymorphic sites of which 37 were parsimony informative, yielding 47 haplotypes from 560 individuals. Of these haplotypes, 38 (80.9%) were found in a single population and 24 were singletons (haplotypes represented by a single sequence) (Table S1). Diversity estimates varied among populations, with the top three highest genetic diversity detected in Ama, Japan (*h *=* *0.871, π *= *0.00140), Yingzuishi, China (*h *=* *0.739, π *= *0.00079), and Ishinomaki, Japan (*h *=* *0.700, π *= *0.00065) (Table 1). Comparatively, genetic diversity in the East China Sea was much lower (*h *=* *0.000–0.562, π *= *0.00000–0.00044) than that in other regions (Table 1).

A total of 592 RuBisCo *rbc*L‐S spacer sequences were obtained with an aligned length of 795 bp with 11 variable sites, representing 14 haplotypes (GenBank accession numbers: KX085135‐KX085148). Of these haplotypes, eight were found in a single population and five were represented by only one sequence. The most abundant haplotype, R1, was shared by 515 specimens, accounting for 86.6% of all samples (Table S1). Diversity indices showed that the populations from Daquan and Nanji Island, China, and Awaji Island, Japan, harbored the highest genetic diversity (*h *=* *0.395–0.455, π *= *0.00058–0.00063), whereas all populations in Korea (POP 7–10) exhibited no genetic diversity (Table 1).

### 3.2 Phylogenetic analysis and population differentiation

The neighbor‐joining tree based on the concatenated *trn*W‐L+*cox*3 sequences revealed substantial phylogeographic structure in *S. fusiforme* (Fig. S1). Three major haplotype groups were discovered, supported by robust bootstrap values (>80%). BI and ML analysis revealed a similar phylogenetic topology as the NJ method (Figure 1). Phylogenetic and network analysis indicated a basic biogeographic pattern of the three genetic groups over space: (i) haplotype group A occurred in the southwest of the Japan‐Pacific ocean; (ii) haplotype group B occurred in the central of the Japan‐Pacific ocean; (iii) haplotype group C occurred in the Sea of Japan and Korea and China coasts (Figure 1a). The main haplotypes of the three groups differed from each other by 8–14 base substitutions (Figure 1b). Group C consisted of three subgroups which distributed in the Sea of Japan (C1), Korea and Yellow–Bohai Sea (C2), and East China Sea (C3), respectively (Figure 1a). The subgroup C1 is paraphyletic and consisted of three clades: H14‐H15, H18‐H23, and monotypic H17. The subgroups C2 and C3 are nested within subgroup C1. The populations in the Sea of Japan (POP 6) and Dongshan Bay, China (POP 25), exhibited a mixture of two subgroups (Figure 1a). Plastid *rbc*L‐S spacer did not reveal substantial phylogenetic structure in *S. fusiforme* (Fig. S2).

**Figure 2:**
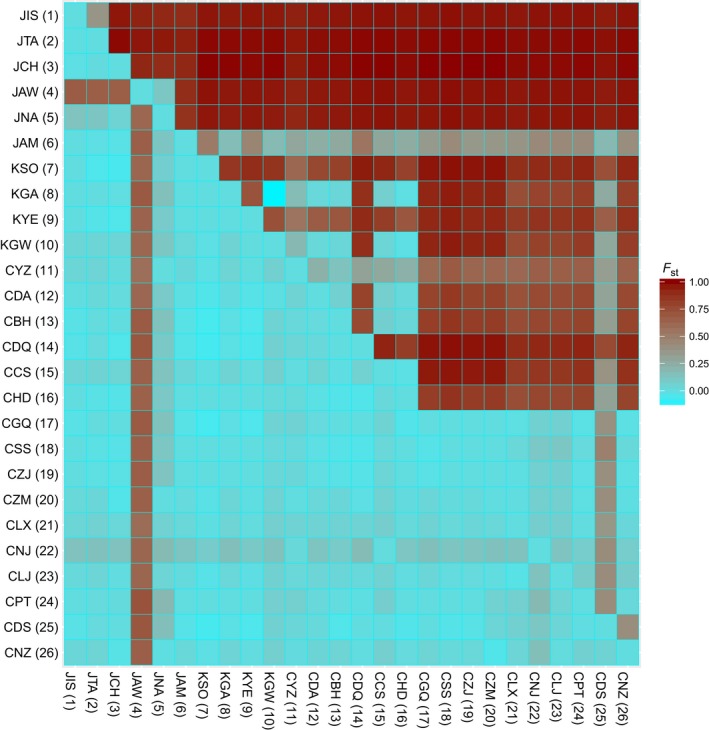
The average *F*_ST_ matrix estimated based on concatenated *trn*W‐L+*cox*3 (upper right) and *rbc*L‐S (lower left) data set, respectively. Dark red and light blue colors indicate high and low *F*_ST_ values, respectively. The abbreviation has been explained in Table 1

Pairwise *F*_ST_ values based on mitochondrial data indicated that populations along the Japan‐Pacific coasts were significantly divergent from all other populations (*F*_ST_ range = 0.865–1.000) (Figure 2, Table S2). The population in the Sea of Japan showed genetic affinity to those along the continental coasts rather than the Japan‐Pacific coasts. Moderate‐to‐high *F*_ST_ values were detected between populations from the Yellow–Bohai Sea (group C2) and East China Sea (group C3) (*F*_ST_ range = 0.299–0.978), but the genetic variance within each marginal sea was low (92% of *F*_ST_ values < 0.300) (Table S2). In contrast, plastid *rbc*L‐S revealed low‐to‐moderate *F*_ST_ values between all populations except for the population in the Awaji Island, Japan (POP 4), with all *F*_ST_ values > 0.60 (Figure 2, Table S2). The AMOVA based on *trn*W‐L+*cox*3 revealed most of the variance (nearly 87.82%) occurred among the groups and/or subgroups (Table S3). The remaining 12.18% variation was found among and within populations, with all *F*‐values statistically significant (Φ_CT_ = 0.8782, Φ_SC_ = 0.5215, Φ_ST_ = 0.9417, *p *<* *.0001 in all cases) (Table S3).

### 3.3 Principal component analysis and divergence time

**Figure 3:**
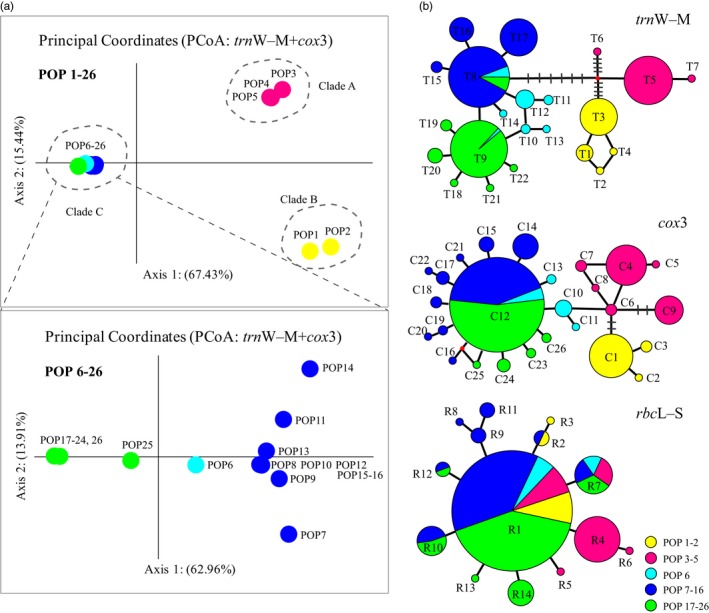
Principal component analysis (PCoA) based on *trn*W‐L+*cox*3 (a) and haplotype network for each marker (b). Each line between main haplotypes represents one mutation step. The groups marked in PCoA are the same as in Figure 1

Deep phylogeographic structure in *S. fusiforme* was also supported by mtDNA‐based principal component analysis (PCoA) (Figure 3a). Firstly, we conducted PCoA with all populations using concatenated mtDNA data and found that *S. fusiforme* populations grouped into three major clusters which were concordant with the groups defined by phylogenetic and network analyses (Figures 1a, 3a). Because there was no clear structure detected within group C (Figures 3a), we then conducted further PCoA analysis for populations (POP 6–26) in this group. As expected, the populations were grouped into three clusters as revealed by phylogenetic analyses (Figures 1a and 3a). Mitochondrial marker‐based haplotype network consistently showed genetic divergence between populations from different regions corresponding to three major groups, while the haplotype network and PCoA inferred from RuBisCo *rbc*L‐S spacer showed no phylogeographic structure (Figure 3b, Fig. S3).

**Figure 4:**
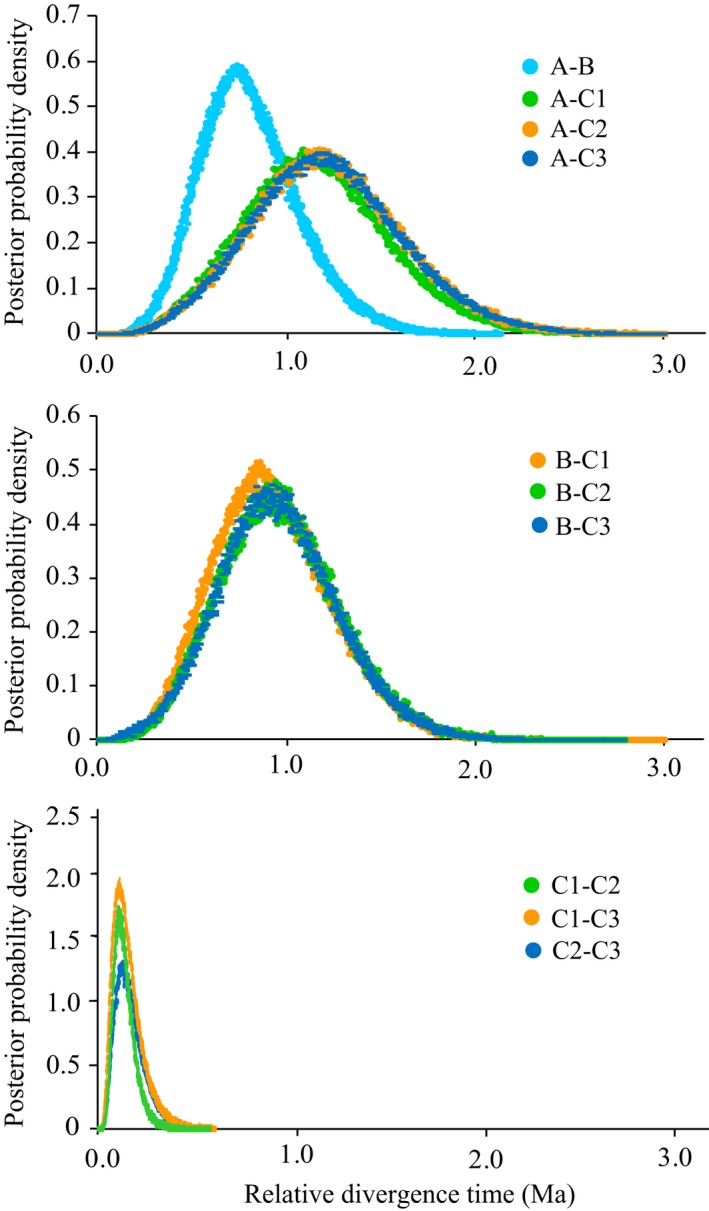
Posterior probability distributions for the relative divergence timescales estimated between groups of *Sargassum thunbergii*

The Ima results indicated that group A may have diverged from group B during the mid‐Pleistocene (*c*. 756 ka, 95%HPD: 273–1,344 ka). The relative divergence time between groups A and C (1,090–1,224 ka) resembled that between groups B and C (858–937 ka) (Figure 4, Table S4). The relative split time between subgroups C1–C3 dated from 106 to 128 ka. In addition, net average sequence distances of mtDNA *cox*3 indicated relative divergence times of 900–2,800 ka between three major groups, whereas the relative divergence times between three subgroups ranged from 0.0 to 100 ka (Table S4).

### 3.4 Demographic history

**Figure 5:**
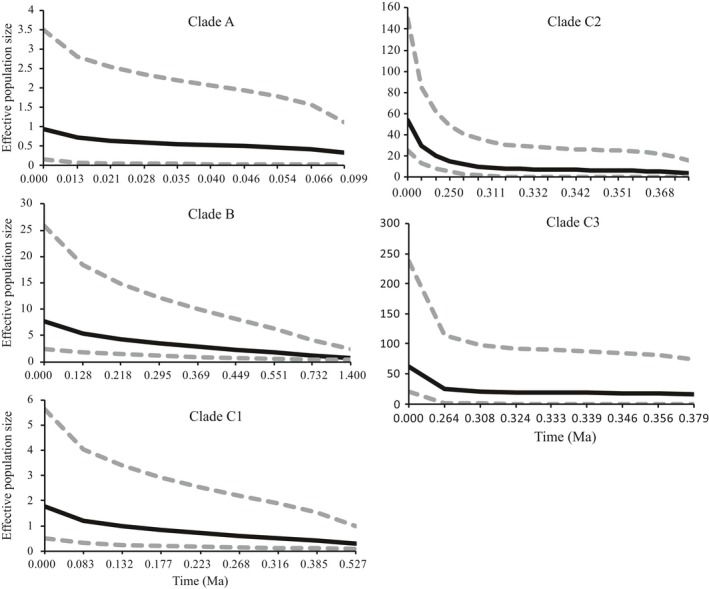
Multilocus extended Bayesian skyline plots (EBSPs) for each group of *Sargassum fusiforme*. Solid lines are the median posterior effective population size through time; dashed lines indicate the 95% highest posterior density interval for each estimate

Neutrality tests showed that both Fu's *F*s and Tajima's *D* were negative for groups C2 and C3, indicating a scenario of historical population expansion (Table S5). For groups A, B, and C1, most of the values were statistically nonsignificant. However, the mismatch distribution for each group showed a unimodal except for group B (Fig. S4), indicating a signal of population expansion. Extended Bayesian skyline plots (EBSPs) analyses detected a slight population expansion in group A and a subsequent population expansion at *c*. 13 ka (Figure 5). The groups B and C1 exhibited a similar demographic trend, with a sudden population expansion at *c*. 83–128 ka, corresponding to the Sangamonian interglacial period. The subgroups C2 and C3 showed a sharp population expansion at *c*. 250–264 ka (Figure 5). The comparison of historical (θ_w_) and contemporary (θ_π_) genetic diversity showed that θw was substantially higher than θ_π_, especially for groups C2 and C3 (Table S5), indicating a recent bottleneck.

## 4 Discussion

### 4.1 Molecular diversity and phylogeographic patterning

In this study, the majority of phylogeographic patterns in *S. fusiforme* were based on the concatenated mitochondrial DNA rather than the plastid *rbc*L‐S. This is principally due to their contrasting phylogeographic resolution (Figure 2, Table S2). Like other oogamous members (e.g., *Fucus*) in the brown algae, the mitochondria and plastid of *Sargassum* are inherited maternally (Motomura, Nagasato, & Kimura, 2010). However, rearrangement and duplication events enable mitochondrial genes to have faster mutation rates than plastids (Mattio & Paryi, 2010; Wan, Wu, Fujihara, & Fang, 2004), leading to different capability in detecting phylogenetic diversity and biogeography of *Sargassum* species (Chan et al., 2013; Cheang et al., 2010; Hu et al., 2011). In general, mitochondrial DNA still provides important insights into biodiversity partitioning and biogeography of *S. fusiforme* in the Asia–Northwest Pacific (ANP) at a fine spatial–temporal scale.

*S. fusiforme* is characterized by deep genetic splits in the ANP (Figure 1 and 3), resembling the phylogeographic structure observed in *S. horneri* (Hu et al., 2011). Such shared structure indicates that both species might have persisted in multiple glacial refugia and phylogeographic patterning is maintained by limited gene flow (Cheang et al., 2010; Hu et al., 2011, 2015; Kim, Hoarau, & Boo, 2012; Kurihara, Horiguchi, Hanyuda, & Kawai, 2016). However, the congeneric *S. polycystum* and *S. thunbergii* in the ANP exhibit high population homogeneity (Chan et al., 2013; Kantachumpoo, Uwai, Noiraksar, & Komatsu, 2014; Li et al., 2016). These contrasting phylogeographic patterns may reflect distinct evolutionary history occurred in *Sargassum* species and/or the physical environment it is subjected to. Interestingly, the population of *S. fusiforme* in the Sea of Japan (JAM) has a mixture of two subgroups (C1 and C2) (Figure 1). It not only harbors the richest diversity, but is also highly divergent from populations in the Japan main islands, rather than populations in China–Korea coasts (Table 1 and Table S2). Biogeographically, the Tsushima Warm Current could bring individuals of *S. fusiforme* (C2) from the southwestern diversity hotspot (e.g., the Jeju Island) into the Sea of Japan (Li et al., 2016), accounting for the structured diversity. Nevertheless, more sampling in the Sea of Japan is necessary to determine whether it could benefit from transregional migration from adjacent areas driven by currents. Likewise, the population in southern China (CDS) also exhibits a mixture of the subgroups (C2 and C3). This diversity pattern likely results from unintentional transportation of C2 from the Yellow–Bohai Sea and/or the Sea of Japan mediated by anthropogenic interference, but further survey is needed.

### 4.2 Conservation insights for *S. fusiforme*

*S. fusiforme* at different geographic scales has shown distinct ecological adaptation to local environmental variables (Kokubu et al., 2015; Nagato & Kawaguchi, 2003; Zou et al., 2006): It is therefore important to determine which populations should be used for breeding and selection purposes in the context of marine culture. The phylogeographic heterogeneity in *S. fusiforme*, with lineages A and B, subgroup C1 distributed around the Japan main islands and subgroups C2 and C3 interspersed along the China coast, respectively (Figure 1), indicates that translocations and long‐term monitoring among these genetically isolated units might be a feasible potion to augment locally depleted/extinct natural resource (Moritz, 1999). In reality, natural populations of *S. fusiforme* in Korea, Japan, and northern China have been collected since 1990s and used as seedlings for farming and cultivation to complement the overexploitation of *S. fusiforme* in Dongtou County, Zhejiang Province, China (Li et al., 2010). It should be noted, however, that these activities could lead to introductions and hybridization that could be harmful for the fitness of progeny and the survival of local populations (outbreeding depression: Frankham, 2005). In addition, the ecological exchangeability among these geographically isolated populations of *S. fusiforme* should be examined prior to conservation approach considering their genetic inexchangeability (Crandall et al., 2000; Moritz, 1999; Rader, Belk, Shiozawa, & Crandall, 2005).

The Japan main islands which host a hotspot of genetic diversity can be explained by the maintenance of distinct ancestral relics and relatively long‐term demographic stability (Dufresnes et al., 2013). The Korea and China coasts with contrasting population homogeneity might stem from interglacial expansion during the late Pleistocene (Stöck et al., 2012), leading to severe loss of variability due to colonization‐associated genetic drift (Excoffier, Foll, & Petit, 2009). These different phylogeographic processes indicate that the lineages of *S. fusiforme* in the Japanese Archipelago are in a more precarious situation than those on the coasts of Korea and China, although each can be identified as an evolutionary significant unit (ESU). Several useful conservation insights can thus be presented for *S. fusiforme*. First, the average *F*_ST_ values among *S. fusiforme* populations in the Japanese Archipelago largely exceed average values between all China–Korea populations, including the population (CNZ) from southern China separated by over 2000 km (Figure 2, Table S2). This mode indicates that populations in the Japanese Archipelago are likely to be especially vulnerable to small population effects and fragmentation (Rossiter, Benda, Dietz, Zhang, & Jones, 2007). In particular, the importance of genetic diversity for historically survived relic populations has long been acknowledged (Frankham, 2005; Rabinowitz & Zeller, 2010). Our results unequivocally show that the south‐to‐central coasts of the Japan main islands have rich groups and endemic diversity. This area might constitute a few refugia during the Pleistocene ice ages (Hu et al., 2011, 2015; Kamei, 1981; Kojima, Fujikura, & Okutani, 2004a), becoming a region that may have been characterized by processes such as habitat fragmentation, range expansion, and secondary contact (Canestrelli, Climmaruta, Costantini, & Nascetti, 2006). Therefore, this region should be identified as a priority for conservation. On the other hand, clustering‐based phylogeny and network analyses (Figure 1 and Table S1) revealed that JAM and CDS are two geographically important sites with lineage admixture (individuals assigned to two different subgroups), and protection of the neighboring populations should help to maximize genetic exchange among lineages.

### 4.3 Broadening conservation effort to marine species in the ANP

The ANP, particularly the Japanese Archipelago, is one of the most prominent marine biodiversity hotspots on the earth (Kerswell, 2006; Marchese, 2015; Norton et al., 1996) and should receive specific conservation priorities to minimize biodiversity loss. For *S. fusiforme*, the structured patterns that high endemic diversity was detected both in the Sea of Japan and along the Pacific–Japan coasts have also been observed in the co‐distributed seaweeds (*S. horneri*, Hu et al., 2011; *Ishige okamurae*, Lee et al., 2012; *Chondrus ocellatus*, Hu et al., 2015), shell (*Turbo* (*Batillus*) *cornutus*, Kojima, Segawa, & Hayashi, 1997; *Batillaria cumingi*, Kojima, Hayashi, Kim, Iijima, & Furota, 2004b; *Cellana nigrolineata*, Nakano, Sasaki, & Kase, 2010), and fish (*Leucopsarion petersii*, Kokita & Nohara, 2011). These analogous phylogeographic patterns in various marine organisms may result from their similar ecological responses to the Quaternary ice ages in the Northern Hemisphere (e.g., long‐term survival in scattered cryptic refugia along southwest Kyushu, Shikoku, and the Kanto district) (Hu et al., 2011; Kamei, 1981; Kojima et al., 2004a) and the variable coastal habitat conditions (Hu et al., 2015). Thus, conservation insights derived from phylogeographic processes of *S. fusiforme* in this study are also applicable to other marine species inhabiting similar coastal environments around the Japanese Archipelago. Integrating ecological niches, biological features, and life histories of different species as a manageable system may minimize the impact of anthropogenic activities and habitat loss on these diversity hotspots, and ultimately to preserve and maintain coastal ecosystem functioning.

### 4.4 Defining evolutionary significant units (ESUs)

As a canopy‐forming macroalga, *S. fusiforme* has a fundamental role in structuring the coastal marine community in the ANP. Conserving this single species will contribute significantly to overall biodiversity and community ecosystems. Our study thus raises the important question whether the diverged genetic lineages in *S. fusiforme* can be defined as conservation units. The concept of ESUs was initially raised in the 1980s by Ryder (1986), and since then, various definitions have been proposed and formulated (Moritz, 1994; Paetkau, 1999). Fraser and Bernatchez (2001) compared the strengths and weaknesses of various criteria to define ESUs using an integrative framework and proposed that ESUs are “a lineage demonstrating highly restricted gene flow from other such lineages within the higher organization level (lineage) of the species.” Here, we adopt the main content of Fraser and Bernatchez's definition and integrate other criteria, including monophyletic mtDNA lineages and the match between genetic features of populations and other features such as geographic distribution and phenotypic distinctiveness (Moritz, 1994). Our concatenated mitochondrial analyses indicate that (i) lineages in *S. fusiforme* are reciprocally monophyletic (Figure 1), (ii) lineages have undergone long‐term historical isolation (Figure 3), (iii) genetic introgression occurred between lineages is negligible (data not shown), and (iv) genetic distinctiveness of lineages matches geographic distribution (Figure 1) and phenotypic traits (e.g., reproduction season and maximum growth period). For example, on the northern coast of Kyushu, Japan, the reproductive activity of *S. fusiforme* restricted to the period from June to August (Nagato & Kawaguchi, 2003), whereas on the coast of Nanao Island, Guangdong, China, its maturation period continued from mid‐April to late June (Zou et al., 2006). Taking these lines of evidence together, the lineages detected in *S. fusiforme* in this study fulfill the requirements of ESUs.

Nevertheless, there are limitations in applying uniparentally inherited mitochondrial genes for conservation biogeography. First of all, the ESUs identified in *S. fusiforme* correspond to species or subspecies boundaries in terms of conservation genetics and taxonomic uncertainties (Karl & Bowen, 1999), and we should be cautious with gene‐specific, lineage‐specific, and population‐specific evolution in mitochondria (Wan et al., 2004). Moreover, Moritz (1994) proposed the following definition of an ESU: “ESUs should be reciprocally monophyletic for mtDNA alleles and show significant divergence of allele frequencies at nuclear loci.” Under this definition, ESUs identified in *S. fusiforme* need to be further examined to check whether there are significant allele frequency shifts in nrDNA, along with the identification of possible elusive phylogeographic processes such as population‐level genetic introgression and incomplete sorting of ancestral genetic polymorphisms (Gaudeul, Gardner, Thomas, Ennos, & Hollingsworth, 2014).

## 5 Concluding Remarks

The structured genetic lineages and sublineages with nearly disjunct geographic boundaries found in *S. fusiforme* reflect both older phylogeographic history and long‐term isolation. These historical phylogeography and cryptic genetic diversity can not only help to understand species’ underlying adaptive potentials following drastic environmental shifts, but also present important insights for conservation purpose and predict how species will respond to future climate change. Nevertheless, ancestral genetic relics have been demonstrated to link to population trends in a biogeographic context (Schmitt & Hewitt, 2004). Geographically, peripheral and central populations have shown variable genetic features and adaptive adjustments to local environments in the coastal marine community (Diekmann & Serräo, 2012; Viejo, Marinez, Arrontes, Astudillo, & Hernández, 2011; Zardi et al., 2015). To maximize conservation effectiveness, research toward linking these evolutionary processes and landscape change to phenotypic performance and fitness emerges as an urgent need to rank lineages of *S. fusiforme* on a scale from less to more susceptible to damage and to recognize protection priorities.

## Data Archiving Statement

GenBank accessions of DNA sequences: KX085135–KX085196. Data for this study are available at https://doi.org/10.5061/dryad.2q6bm.
