# Supplementary material for: Hidden diversity and phylogeographic history provide conservation insights for the edible seaweed Sargassum fusiforme in the Northwest Pacific
Source: Evol Appl. 2017 Feb 19;10(4):366–78. doi: 10.1111/eva.12455 (PMC5367075; doi:10.1111/eva.12455)

**Table S1** Haplotype distribution in each *Sargassum fusiforme* population. Haplotypes inferred from *trn*W-L+*cox*3 and *rbc*L-S were identified by the capital H or R. Population codes in parentheses are the same as in Table 1 and Fig. 1.

| Sampling localities | *trn*W–M+*cox*3 | *rbc*L–S |
| --- | --- | --- |
| 1. Ishinomaki, Miyagi, Japan (JIS) | H1(7),H2(1),H3(6),H4(1),H5(1) | R1(16) |
| 2. Tateyama Bay, Chiba, Japan (JTA) | H3(27),H6(3) | R1(28),R2(1),R3(1) |
| 3. Chita, Aichi, Japan (JCH) | H7(19) | R1(19),R7(1) |
| 4. Awaji Island, Hyogo, Japan (JAW) | H8(22),H9(1),H10(1) | R1(5),R4(23),R5(1),R6(1) |
| 5. Naruto, Tokushima, Japan (JNA) | H8(10),H11(2),H12(3),H13(1) | R1(16),R7(4) |
| 6. Ama,Shimane, Japan (JAM) | H14(5),H15(1),H16(7),H17(1),H18(1),H19(1),H20(1),H21(5),H22(1),H23(2),H24(1) | R1(26),R7(3) |
| 7. Sokcho, Gangwondo, Korea (KSO) | H25(14), H26(1) | R1(14) |
| 8. Gauido, Chungcheongnamdo, Korea (KGA) | H16(7),H27(1) | R1(8) |
| 9. Yeongsando, Jeollanamdo, Korea (KYE) | H16(3),H28(16) | R1(19) |
| 10. Gwanmaedo, Jeollanamdo, Korea (KGW) | H16(9),H27(1) | R1(10) |
| 11. Yingzuishi, Liaoning, China (CYZ) | H16(9),H29(2),H30(10),H31(1),H32(3),H33(1) | R1(21),R8(1),R9(2) |
| 12. Daquan, Liaoningn, China (CDA) | H16(18),H29(2),H34(3) | R1(19),R2(2),R7(3),R10(3) |
| 13. Beihuangcheng, Yantai, China (CBH) | H16(24),H29(1),H30(4) | R1(26),R11(2),R12(1) |
| 14. Daqin, Yantai, China (CDQ) | H30(19),H35(1) | R1(23),R10(1) |
| 15. Chengshantou, Weihai, China (CCS) | H16(27),H32(1) | R1(29),R7(1) |
| 16. Huidao, Weihai, China (CHD) | H16(23),H29(1),H36(1),H37(1) | R1(24),R10(3) |
| 17. Gouqi Island, Zhejiang, China (CGQ) | H38(12) | R1(12) |
| 18. Shengsi, Zhejaing, China (CSS) | H38(24) | R1(23),R13(1) |
| 19. Zhujiajian, Zhejiang, China (CZJ) | H38(13) | R1(13) |
| 20. Zhumen, Zhejiang, China (CZM) | H38(14) | R1(15) |
| 21. Luxi Island, Zhejiang, China (CLX) | H38(18),H39(1),H40(1),H41(1) | R1(20),R7(2) |
| 22. Nanji Island, Zhejiang, China (CNJ) | H38(27),H42(5) | R1(26),R7(2),R12(1),R14(5) |
| 23. Lianjiang, Fujian, China (CLJ) | H38(26),H43(5) | R1(24),R10(7) |
| 24. Putian, Fujian, China (CPT) | H38(25),H41(2),H44(1) | R1(25),R7(2),R14(3) |
| 25. Dongshan Bay, Fujian, China (CDS) | H16(12),H38(11),H45(1) | R1(27) |
| 26. Naozhou, Guangdong, China (CNZ) | H38(23),H46(1),H47(2) | R1(27),R10(1) |

**Table S2** Pairwise *F*_ST_ estimates among *Sargassum fusiforme* populations based on mitochondrial *trn*W-L+*cox*3 (Mt) and plastid *rbc*L–S (Cp). Statistically significant *F*_ST_ estimates are marked in bold (*P* < 0.05). Population codes are the same as in Table S1.

| **Mt** | JIS | JTA | JCH | JAW | JNA | JAM | KSO | KGA | KYE | KGW | CYZ | CDA | CBH | CDQ | CCS | CHD | CGQ | CSS | CZJ | CZM | CLX | CNJ | CLJ | CPT | CDS | CNZ |
| --- | --- | --- | --- | --- | --- | --- | --- | --- | --- | --- | --- | --- | --- | --- | --- | --- | --- | --- | --- | --- | --- | --- | --- | --- | --- | --- |
| JIS | 0.000 |  |  |  |  |  |  |  |  |  |  |  |  |  |  |  |  |  |  |  |  |  |  |  |  |  |
| JTA | **0.378** | 0.000 |  |  |  |  |  |  |  |  |  |  |  |  |  |  |  |  |  |  |  |  |  |  |  |  |
| JCH | **0.958** | **0.987** | 0.000 |  |  |  |  |  |  |  |  |  |  |  |  |  |  |  |  |  |  |  |  |  |  |  |
| JAW | **0.926** | **0.961** | **0.914** | 0.000 |  |  |  |  |  |  |  |  |  |  |  |  |  |  |  |  |  |  |  |  |  |  |
| JNA | **0.908** | **0.954** | **0.891** | **0.116** | 0.000 |  |  |  |  |  |  |  |  |  |  |  |  |  |  |  |  |  |  |  |  |  |
| JAM | **0.894** | **0.933** | **0.906** | **0.886** | **0.865** | 0.000 |  |  |  |  |  |  |  |  |  |  |  |  |  |  |  |  |  |  |  |  |
| KSO | **0.964** | **0.989** | **0.995** | **0.968** | **0.961** | **0.515** | 0.000 |  |  |  |  |  |  |  |  |  |  |  |  |  |  |  |  |  |  |  |
| KGA | **0.951** | **0.986** | **0.994** | **0.957** | **0.944** | **0.166** | **0.851** | 0.000 |  |  |  |  |  |  |  |  |  |  |  |  |  |  |  |  |  |  |
| KYE | **0.962** | **0.985** | **0.988** | **0.963** | **0.956** | **0.465** | **0.887** | **0.720** | 0.000 |  |  |  |  |  |  |  |  |  |  |  |  |  |  |  |  |  |
| KGW | **0.954** | **0.987** | **0.994** | **0.960** | **0.948** | **0.184** | **0.861** | **-**0.123 | **0.734** | 0.000 |  |  |  |  |  |  |  |  |  |  |  |  |  |  |  |  |
| CYZ | **0.930** | **0.959** | **0.946** | **0.926** | **0.912** | **0.288** | **0.619** | **0.171** | **0.551** | **0.187** | 0.000 |  |  |  |  |  |  |  |  |  |  |  |  |  |  |  |
| CDA | **0.957** | **0.980** | **0.980** | **0.956** | **0.948** | **0.247** | **0.772** | 0.025 | **0.673** | 0.028 | **0.227** | 0.000 |  |  |  |  |  |  |  |  |  |  |  |  |  |  |
| CBH | **0.963** | **0.983** | **0.983** | **0.961** | **0.955** | **0.273** | **0.799** | 0.037 | **0.704** | 0.037 | **0.137** | 0.066 | 0.000 |  |  |  |  |  |  |  |  |  |  |  |  |  |
| CDQ | **0.969** | **0.990** | **0.996** | **0.972** | **0.966** | **0.547** | **0.946** | **0.878** | **0.901** | **0.884** | **0.309** | **0.794** | **0.764** | 0.000 |  |  |  |  |  |  |  |  |  |  |  |  |
| CCS | **0.973** | **0.991** | **0.996** | **0.974** | **0.970** | **0.287** | **0.915** | 0.071 | **0.819** | 0.039 | **0.277** | 0.072 | 0.070 | **0.923** | 0.000 |  |  |  |  |  |  |  |  |  |  |  |
| CHD | **0.962** | **0.983** | **0.984** | **0.961** | **0.954** | **0.256** | **0.803** | -0.009 | **0.703** | -0.012 | **0.229** | 0.026 | 0.037 | **0.821** | -0.013 | 0.000 |  |  |  |  |  |  |  |  |  |  |
| CGQ | **0.965** | **0.991** | **1.000** | **0.970** | **0.962** | **0.352** | **0.964** | **0.912** | **0.907** | **0.918** | **0.613** | **0.789** | **0.816** | **0.969** | **0.952** | **0.823** | 0.000 |  |  |  |  |  |  |  |  |  |
| CSS | **0.976** | **0.993** | **1.000** | **0.978** | **0.974** | **0.433** | **0.975** | **0.945** | **0.933** | **0.947** | **0.683** | **0.838** | **0.854** | **0.978** | **0.963** | **0.862** | 0.000 | 0.000 |  |  |  |  |  |  |  |  |
| CZJ | **0.966** | **0.991** | **1.000** | **0.971** | **0.964** | **0.360** | **0.965** | **0.916** | **0.910** | **0.921** | **0.620** | **0.795** | **0.820** | **0.970** | **0.953** | **0.827** | 0.000 | 0.000 | 0.000 |  |  |  |  |  |  |  |
| CZM | **0.968** | **0.992** | **1.000** | **0.972** | **0.965** | **0.368** | **0.967** | **0.920** | **0.912** | **0.925** | **0.627** | **0.799** | **0.824** | **0.971** | **0.954** | **0.831** | 0.000 | 0.000 | 0.000 | 0.000 |  |  |  |  |  |  |
| CLX | **0.960** | **0.983** | **0.984** | **0.961** | **0.953** | **0.383** | **0.880** | **0.746** | **0.839** | **0.759** | **0.614** | **0.725** | **0.751** | **0.893** | **0.835** | **0.749** | -0.009 | 0.033 | -0.004 | **0.001** | 0.000 |  |  |  |  |  |
| CNJ | **0.968** | **0.985** | **0.986** | **0.967** | **0.962** | **0.441** | **0.898** | **0.792** | **0.862** | **0.799** | **0.662** | **0.760** | **0.780** | **0.907** | **0.851** | **0.780** | 0.060 | 0.107 | 0.066 | **0.071** | **0.076** | 0.000 |  |  |  |  |
| CLJ | **0.967** | **0.985** | **0.986** | **0.967** | **0.962** | **0.437** | **0.897** | **0.788** | **0.860** | **0.796** | **0.658** | **0.758** | **0.777** | **0.906** | **0.849** | **0.778** | 0.065 | 0.113 | 0.070 | 0.075 | **0.079** | **0.131** | 0.000 |  |  |  |
| CPT | **0.969** | **0.987** | **0.990** | **0.970** | **0.965** | **0.428** | **0.916** | **0.822** | **0.878** | **0.829** | **0.660** | **0.775** | **0.795** | **0.924** | **0.877** | **0.796** | -0.017 | 0.018 | -0.012 | -0.008 | 0.006 | 0.085 | 0.089 | 0.000 |  |  |
| CDS | **0.949** | **0.975** | **0.971** | **0.948** | **0.938** | **0.189** | **0.738** | **0.262** | **0.661** | **0.277** | **0.330** | **0.299** | **0.326** | **0.762** | **0.389** | **0.308** | **0.399** | **0.484** | **0.407** | **0.416** | **0.367** | **0.425** | **0.422** | **0.424** | 0.000 |  |
| CNZ | **0.968** | **0.987** | **0.989** | **0.968** | **0.963** | **0.418** | **0.913** | **0.814** | **0.873** | **0.821** | **0.651** | **0.768** | **0.789** | **0.921** | **0.874** | **0.790** | -0.014 | 0.023 | -0.009 | -0.005 | **0.029** | **0.084** | **0.087** | **0.026** | **0.414** | 0.000 |

| **Cp** | JIS | JTA | JCH | JAW | JNA | JAM | KSO | KGA | KYE | KGW | CYZ | CDA | CBH | CDQ | CCS | CHD | CGQ | CSS | CZJ | CZM | CLX | CNJ | CLJ | CPT | CDS | CNZ |
| --- | --- | --- | --- | --- | --- | --- | --- | --- | --- | --- | --- | --- | --- | --- | --- | --- | --- | --- | --- | --- | --- | --- | --- | --- | --- | --- |
| JIS | 0.000 |  |  |  |  |  |  |  |  |  |  |  |  |  |  |  |  |  |  |  |  |  |  |  |  |  |
| JTA | -0.005 | 0.000 |  |  |  |  |  |  |  |  |  |  |  |  |  |  |  |  |  |  |  |  |  |  |  |  |
| JCH | -0.012 | 0.009 | 0.000 |  |  |  |  |  |  |  |  |  |  |  |  |  |  |  |  |  |  |  |  |  |  |  |
| JAW | **0.672** | **0.659** | **0.664** | 0.000 |  |  |  |  |  |  |  |  |  |  |  |  |  |  |  |  |  |  |  |  |  |  |
| JNA | 0.135 | **0.122** | 0.050 | **0.616** | 0.000 |  |  |  |  |  |  |  |  |  |  |  |  |  |  |  |  |  |  |  |  |  |
| JAM | 0.000 | -0.011 | -0.019 | **0.661** | 0.122 | 0.000 |  |  |  |  |  |  |  |  |  |  |  |  |  |  |  |  |  |  |  |  |
| KSO | 0.000 | -0.046 | -0.056 | **0.621** | 0.070 | 0.000 | 0.000 |  |  |  |  |  |  |  |  |  |  |  |  |  |  |  |  |  |  |  |
| KGA | 0.000 | 0.003 | -0.003 | **0.687** | 0.153 | 0.000 | 0.000 | 0.000 |  |  |  |  |  |  |  |  |  |  |  |  |  |  |  |  |  |  |
| KYE | 0.000 | -0.030 | -0.040 | **0.636** | 0.091 | 0.000 | 0.000 | 0.000 | 0.000 |  |  |  |  |  |  |  |  |  |  |  |  |  |  |  |  |  |
| KGW | 0.038 | 0.042 | 0.044 | **0.619** | **0.116** | 0.030 | -0.011 | 0.050 | 0.007 | 0.000 |  |  |  |  |  |  |  |  |  |  |  |  |  |  |  |  |
| CYZ | 0.038 | **0.046** | 0.014 | **0.576** | 0.007 | 0.029 | -0.010 | 0.048 | 0.007 | **0.066** | 0.000 |  |  |  |  |  |  |  |  |  |  |  |  |  |  |  |
| CDA | 0.001 | 0.027 | 0.013 | **0.614** | **0.098** | -0.006 | -0.041 | 0.009 | -0.026 | -0.005 | **0.054** | 0.000 |  |  |  |  |  |  |  |  |  |  |  |  |  |  |
| CBH | -0.018 | 0.012 | 0.001 | **0.681** | **0.142** | -0.025 | -0.060 | -0.010 | -0.044 | 0.052 | 0.026 | 0.018 | 0.000 |  |  |  |  |  |  |  |  |  |  |  |  |  |
| CDQ | -0.023 | 0.017 | -0.040 | **0.705** | 0.106 | -0.029 | -0.062 | -0.016 | -0.047 | 0.064 | 0.043 | 0.025 | 0.001 | 0.000 |  |  |  |  |  |  |  |  |  |  |  |  |
| CCS | 0.043 | 0.052 | 0.047 | **0.653** | **0.136** | 0.035 | -0.005 | 0.054 | 0.012 | **0.072** | 0.005 | 0.047 | -0.007 | 0.063 | 0.000 |  |  |  |  |  |  |  |  |  |  |  |
| CHD | 0.000 | -0.019 | -0.028 | **0.649** | 0.108 | 0.000 | 0.000 | 0.000 | 0.000 | 0.019 | 0.019 | -0.015 | -0.033 | -0.037 | 0.025 | 0.000 |  |  |  |  |  |  |  |  |  |  |
| CGQ | -0.018 | 0.012 | 0.001 | **0.681** | **0.142** | -0.025 | -0.060 | -0.010 | -0.044 | 0.052 | 0.056 | 0.018 | 0.000 | 0.001 | 0.053 | -0.033 | 0.000 |  |  |  |  |  |  |  |  |  |
| CSS | 0.000 | -0.015 | -0.023 | **0.655** | 0.115 | 0.000 | 0.000 | 0.000 | 0.000 | 0.025 | 0.025 | -0.010 | -0.028 | -0.033 | 0.030 | 0.000 | -0.028 | 0.000 |  |  |  |  |  |  |  |  |
| CZJ | 0.000 | -0.008 | -0.015 | **0.667** | **0.129** | 0.000 | 0.000 | 0.000 | 0.000 | 0.034 | 0.034 | -0.003 | -0.021 | -0.026 | 0.039 | 0.000 | -0.021 | 0.000 | 0.000 |  |  |  |  |  |  |  |
| CZM | 0.027 | 0.034 | -0.037 | **0.651** | 0.000 | 0.018 | -0.022 | 0.038 | -0.005 | 0.057 | 0.002 | 0.030 | 0.034 | -0.010 | 0.063 | 0.008 | 0.034 | 0.014 | 0.023 | 0.000 |  |  |  |  |  |  |
| CLX | 0.040 | **0.063** | 0.032 | **0.593** | **0.063** | 0.032 | -0.004 | 0.049 | 0.012 | **0.072** | 0.051 | **0.054** | **0.057** | **0.054** | **0.076** | 0.023 | **0.057** | 0.028 | 0.036 | 0.033 | 0.000 |  |  |  |  |  |
| CNJ | 0.141 | **0.145** | **0.141** | **0.623** | **0.179** | 0.131 | 0.087 | **0.155** | 0.105 | **0.141** | **0.027** | **0.126** | 0.094 | **0.172** | 0.011 | 0.119 | **0.154** | 0.125 | 0.136 | **0.145** | **0.140** | 0.000 |  |  |  |  |
| CLJ | 0.021 | **0.043** | 0.003 | **0.623** | 0.042 | 0.014 | -0.023 | 0.030 | -0.007 | **0.060** | 0.031 | **0.042** | 0.037 | 0.024 | **0.063** | 0.005 | 0.037 | 0.010 | 0.018 | 0.002 | -0.024 | **0.138** | 0.000 |  |  |  |
| CPT | 0.000 | 0.018 | 0.015 | **0.721** | **0.192** | 0.000 | 0.000 | 0.000 | 0.000 | 0.074 | 0.071 | 0.025 | 0.005 | -0.004 | 0.077 | 0.000 | 0.005 | 0.000 | 0.000 | 0.062 | **0.069** | 0.186 | 0.049 | 0.000 |  |  |
| CDS | -0.022 | 0.016 | 0.003 | **0.698** | **0.156** | -0.028 | -0.061 | -0.015 | -0.046 | 0.060 | **0.037** | 0.023 | -0.040 | 0.000 | 0.005 | -0.036 | 0.000 | -0.031 | -0.025 | 0.040 | **0.064** | 0.113 | 0.043 | -0.001 | 0.000 |  |
| CNZ | 0.036 | 0.048 | -0.024 | **0.660** | -0.005 | 0.028 | -0.010 | 0.046 | 0.006 | **0.071** | 0.008 | 0.045 | 0.047 | 0.004 | **0.074** | 0.018 | 0.047 | 0.023 | 0.032 | -0.041 | 0.042 | 0.158 | 0.011 | 0.067 | 0.052 | 0.000 |

**Table S3** Analysis of molecular variance (AMOVA) to partition genetic variance in the Northwest Pacific *Sargassum fusiforme* based on *trn*W-L+*cox*3

| Markers | Source of variation | d.f. | %var | *F* values |
| --- | --- | --- | --- | --- |
|  | Among clades | 4 | 87.82 | Φ_CT_ = 0.8782*** |
|  | Among populations within clades | 21 | 6.35 | Φ_SC_ = 0.5215*** |
|  | Within populations | 534 | 5.83 | Φ_ST_ = 0.9417*** |

^***^*P* < 0.0001; n.s. not significant

**Table S4** Relative divergence time estimated between clades. The values in parentheses are 95% highest posterior density (HPD) intervals for IMa analyses.

| Clades | Clade A | Clade B | Clade C1 | Clade C2 | Clade C3 |
| --- | --- | --- | --- | --- | --- |
| IMa |  |  |  |  |  |
| Clade A | - |  |  |  |  |
| Clade B | 0.756 (0.273–1.344) | - |  |  |  |
| Clade C1 | 1.090 (0.404–1.846) | 0.858 (0.313–1.488) | - |  |  |
| Clade C2 | 1.224 (0.410–2.016) | 0.937 (0.361–1.615) | 0.106 (0.043–0.245) | - |  |
| Clade C3 | 1.176 (0.286–2.038) | 0.923 (0.330–1.641) | 0.113 (0.034–0.313) | 0.128 (0.043–0.321) | - |
| *d*A = *d*XY-(*d*X + *d*Y)/2 | | |  |  |  |
| Clade A | - |  |  |  |  |
| Clade B | 1.200–1.800 | - |  |  |  |
| Clade C1 | 0.900–1.350 | 1.533–2.300 | - |  |  |
| Clade C2 | 1.200–1.800 | 1.867–2.800 | 0.067–0.100 | - |  |
| Clade C3 | 1.200–1.800 | 1.867–2.800 | 0.067–0.100 | 0.000 | - |
|  | | | | | |

**Table S5** Neutrality tests and demographic estimates based on mitochondrial *trn*W-L+*cox*3 and platid *rbc*L-S data. *Θ*π and *θ*w are current and historical estimates of genetic diversities, respectively.

|  |  | *θ*π | *θ*w | Tajima’s *D* | Fu’s *F*s |
| --- | --- | --- | --- | --- | --- |
| *trn*W–M+*cox*3 | Clade A | 0.00124 | 0.00156 | -0.589^n.s.^ | -0.007^n.s.^ |
|  | Clade B | 0.00040 | 0.00066 | -0.923^n.s.^ | -3.054^*^ |
|  | Clade C1 | 0.00157 | 0.00148 | 0.203^n.s.^ | -3.377^*^ |
|  | Clade C2 | 0.00054 | 0.00158 | -1.60^*^ | -12.762^***^ |
|  | Clade C3 | 0.00014 | 0.00098 | -1.886^*^ | -11.871^***^ |
| *rbc*L–S | Clade A | 0.00082 | 0.00104 | -0.462^n.s.^ | -1.01^n.s.^ |
|  | Clade B | 0.00016 | 0.00057 | -1.307^n.s.^ | -2.207^*^ |
|  | Clade C1 | 0.00032 | 0.00035 | -0.133^n.s.^ | 0.341^n.s.^ |
|  | Clade C2 | 0.00022 | 0.00146 | -1.77^**^ | -9.785^***^ |
|  | Clade C3 | 0.00026 | 0.00105 | -1.42^*^ | -5.110^*^ |

^*^*P* < 0.05; ^**^*P* < 0.001; ^***^*P* < 0.0001; n.s. not signiﬁcant.

**Figure S1**. The neighbour-joining tree inferred from concatenated mitochondrial data. Bootstrap supports >50 are indicated around branches.


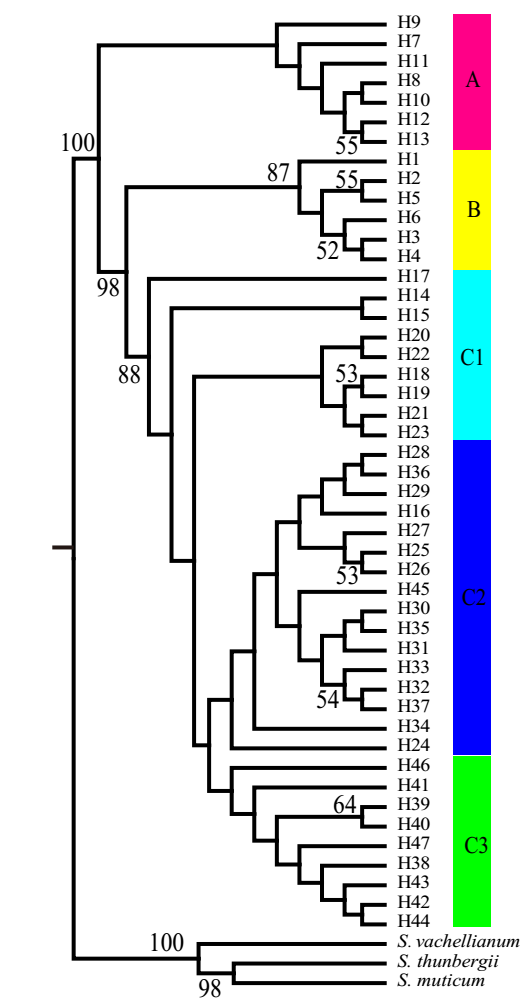


Figure S2. The maximum-likelihood (ML)/Bayesian inference (BI) trees inferred from plastid *rbc*L-S spacer. Bootstrap values >50 for ML (upper) and BI (lower) are indicated around branches.


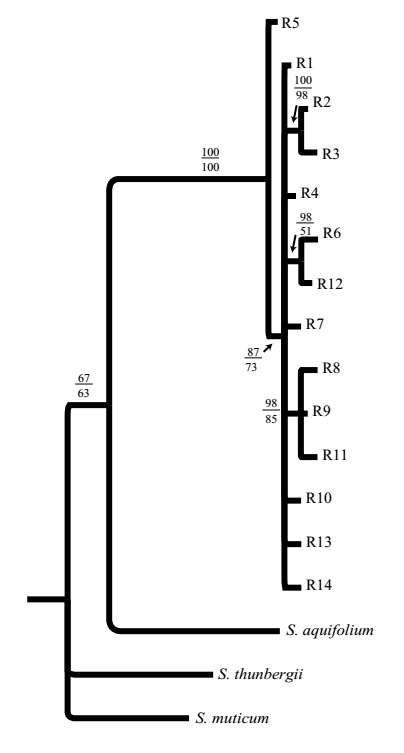


Figure S3. Principal component analysis (PCoA) based on plastid *rbc*L-S spacer. The groups marked in PCoA are the same as in Fig. 1.


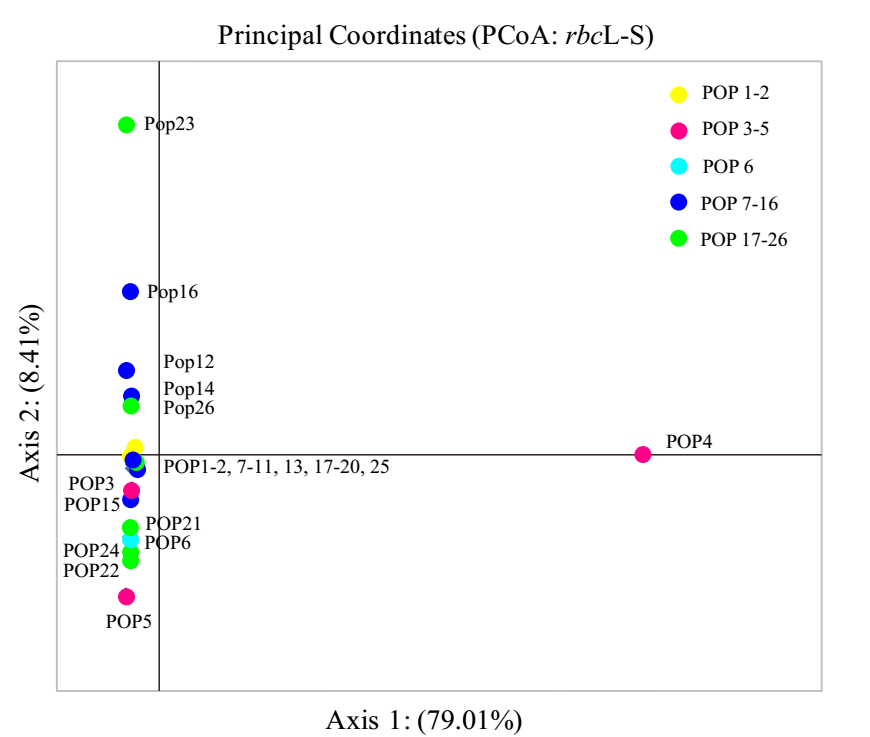


Figure S4. Mismatch distribution of the five clades/sub-clades in *Sargassum fusiforme* based on (a) *trn*W-L and (b) *rbc*L-S data sets. Histograms represent observed frequencies and lines represent expected frequencies.


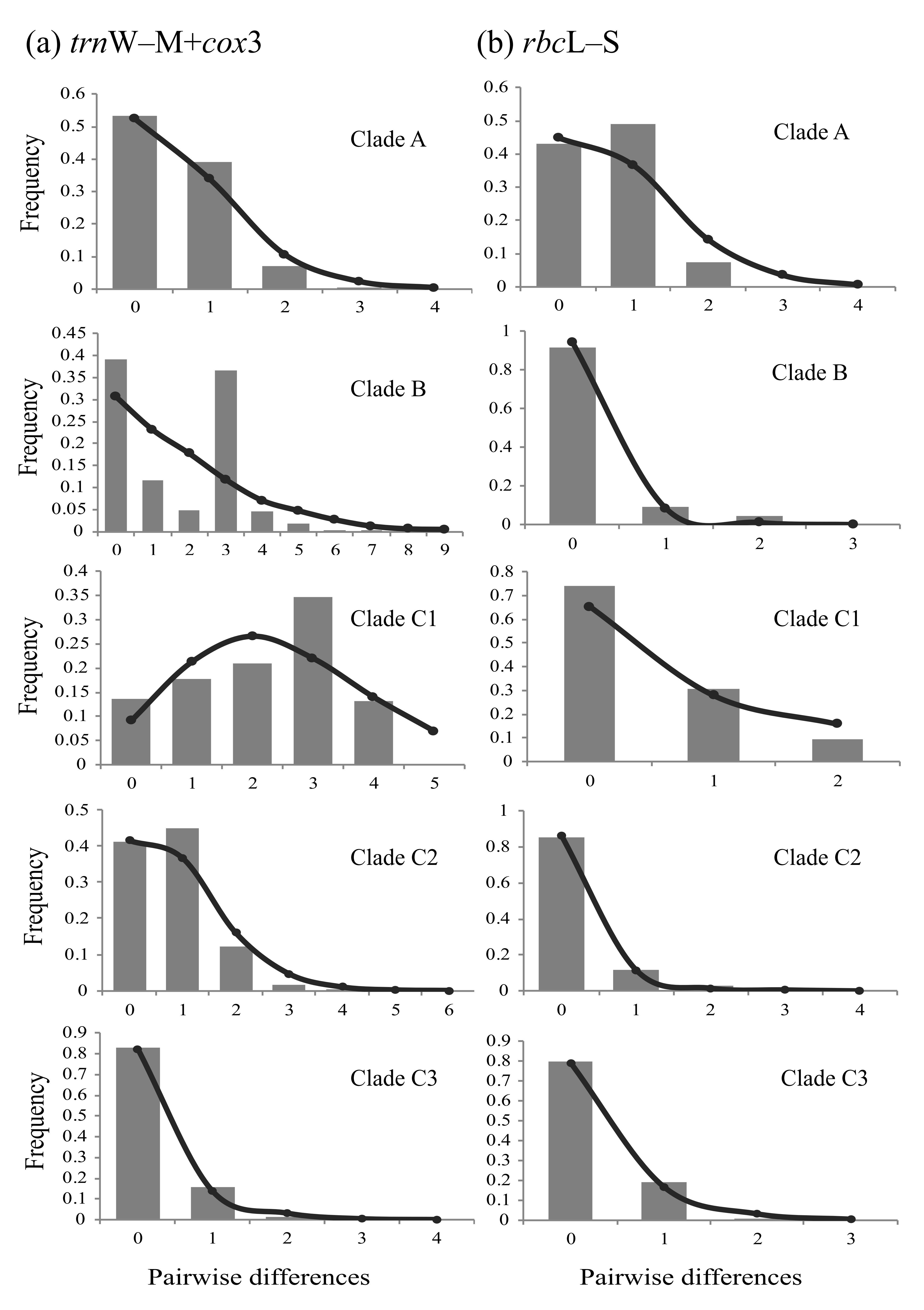

Supplement: Supplementary file 1 [file EVA-10-366-s001.docx]
